# Memory-Enhancing Effect of 8-Week Consumption of the Quercetin-Enriched Culinary Herbs-Derived Functional Ingredients: A Randomized, Double-Blind, Placebo-Controlled Clinical Trial

**DOI:** 10.3390/foods11172678

**Published:** 2022-09-02

**Authors:** Jintanaporn Wattanathorn, Woraluck Somboonporn, Wipawee Thukham-Mee, Sudarat Sungkamnee

**Affiliations:** 1 Department of Physiology, Faculty of Medicine, Research Institute of High Human Performance and Health Promotion, Khon Kaen University, Khon Kaen 40002, Thailand; 2 Department of Obstetrics and Gynecology, Faculty of Medicine, Khon Kaen University, Khon Kaen 40002, Thailand

**Keywords:** memory, *Polygonum odorarum*, *Morus alba*

## Abstract

Due to great demand for memory enhancers, the memory-enhancing effects and the possible underlying mechanisms of the functional ingredients derived from the combined extract of *Polygonum odoratum* and *Morus alba* were investigated. A total of 45 participants randomly received either a placebo or the developed herbal supplement at a dose of 50 or 1500 mg/day. The consumption was done once daily for 8 weeks. Working memory was assessed via both an event-related potential and computerized battery tests at baseline and at the end of the 8-week study period. Acetylcholinesterase (AChE) and monoamine oxidase type A and type B (MAO-A, MAO-B) levels were also measured at the end of the study. The subjects who consumed the supplement containing a developed functional ingredient at a dose of 1500 mg/day showed reduced latencies but increased amplitudes of N100 and P300. An improvement in working memory and the suppression of AChE, MAO-A, and MAO-B activities were also observed. Therefore, this study clearly demonstrates the cognitive enhancing effect of the developed herbal congee, which may be associated with the suppressions of AChE and both types of MAO.

## 1. Introduction

Currently, the demand for functional food ingredients or substances that provide health benefits beyond basic nutrition is growing fast due to an increase in the elderly population, health awareness of the consumer, and a paradigm shift in health to prevention medicine. It has been reported that functional food ingredients have gained much attention from the food and beverage industry [1]. Due to the growing popularity of food and beverage supplements aimed at memory enhancement, functional ingredients that can enhance and maintain memory are in great demand [2].

Memory, especially working memory, or the ability to maintain and manipulate information over a period [3], is necessary for daily life skills such as reading, comprehension, and problem-solving [4]. This capacity declines as age advances [5]. Normal age-related memory decline is modest, but severe memory decline causes memory impairment. Currently, we can monitor working memory by using event-related potential (ERP), a measurement of brain activity or neuronal biomarkers related to brain events, including working memory [6,7,8,9,10,11]. It is regarded as a good index for measuring cognitive function by providing the time course of information processing, including expectancy, attention, cognition search, decision making, and memorization [12,13]. The N100 component is a negative deflection of brain activity that occurs between 65 and 135 ms after the onset of an auditory stimulus and is largely distributed over the fronto-central regions of the brain, whereas the P300 component is a positive deflection of brain activity that can be observed between 280 and 350 ms after the onset of stimulus and is primarily distributed over the central–parietal region of the brain [14,15]. It has been reported that N100 reflects attention activation [14,16], whereas P300 reflects information processing [17]. The latency of a brain wave indicates neural speed or brain efficiency, whereas the amplitude of the wave indicates neural power or cognitive resources [18]. In addition to ERP, cognitive function, including working memory, can also be measured by standardized neuropsychological test batteries [19,20,21].

Memory impairment is recognized as one of the important problems in menopause [22], especially during the transition period. Around 49% of menopausal women are subjected to cognitive changes [23]. It has been demonstrated that subjective complaints are associated with working memory and attention performance as well as depression, anxiety, somatic complaints, and sleep disturbance [22]. These symptoms are reported to be associated with the level of estrogen [24,25]. In addition, several studies reveal that estrogen replacement therapy can improve the function of the frontal lobe and working memory [26] by modulating the functions of the cholinergic [27] and monoaminergic [25] systems. However, long-term treatment with estrogen replacement therapy can impair frontal lobe functions [28,29]. In addition, it is also associated with increased risks of breast cancer, stroke, coronary heart disease, pulmonary embolism, and uterine bleeding [30,31,32,33]. Therefore, a novel strategy to prevent cognitive impairment in menopausal women is still required.

Multiple lines of evidence have demonstrated that polyphenol substances enhance cognitive function in healthy adults [34]. Polyphenol-rich extracts of culinary herbs such as *phak phai* or Vietnamese coriander (*Polygonum odoratum*) and *mohn* or *Morus alba* also show neuroprotective and cognition-enhancing effects [35,36,37]. In addition, it has been revealed that the polyherbal recipe can provide benefits via synergistic interactions, leading to an increase in efficiency, a reduction in undesirable effects, an increase in stability or bioavailability, and a reduction of therapeutic dose [38]. Our previous study clearly demonstrated that the functional ingredient derived from the combined extract of *P. odoratum* and *M. alba* is safe to consume, without toxicity and side effects [39]. Owing to the memory-enhancing effects of both culinary herbs mentioned earlier and the synergistic interaction concept, it was hypothesized that functional ingredients containing the combined extract of *P. odoratum* and *M. alba* should enhance the cognitive function of peri- and postmenopausal women. Due to a lack of supporting evidence in clinical trial studies, this study was carried out to elucidate this issue. The possible underlying mechanisms were also further explored.

## 2. Materials and Methods

### 2.1. Preparation of the Culinary Herbs-Derived Functional Ingredients (MP)

The functional ingredient used in this study was prepared from the combined extract of phak phai or phak prew, or *Polygonum odoratum*, and mohn or *Morus alba*, two culinary herbs consumed widely in Thai cuisine, particularly in the north-east region of Thailand. The details have been mentioned elsewhere [39,40]. In brief, aerial parts of *P. odoratum* and leaves of *Morus alba* from Khon Kaen province were authenticated and prepared as a water extract by using the decoction method. Then, they were mixed with an appropriate ratio (petty patent number 9314, Department of Intellectual Property, Thailand). The final functional ingredient contained quercetin at a concentration of 22.846 mg quercetin equivalent (QE)/100 mg [39].

In this study, the developed functional ingredient was added to a congee-based formula at doses of 50 and 1500 mg/serving, which provided around 95.50 and 97.25 Kcal of energy, respectively. The placebo was also prepared using the same formulas, but no functional ingredient was added. The energy provided by each serving of placebo was 94.24 Kcal. The ingredients were described in detail by Wattanathorn et al., 2018 [39], and the fingerprint chromatogram was previously shown elsewhere [39].

### 2.2. Study Design

This study was performed as a randomized, double-blind, placebo-controlled trial. It was registered on ClinicalTrials.gov (Identifier NCT02563374) and was conducted in accordance with the International Conference of Harmonization (ICH) for Good Clinical Practice (GCP) and in compliance with the Declaration of Helsinki and its further amendments. All protocols were reviewed and approved by the Khon Kaen University Ethical Committee on Human Research (HE571373).

The study is an 8-week intervention period consisting of 2 visits: a baseline visit (prior to an intervention) and an 8-week consumption period of a supplement containing the functional ingredient derived from the combined extract of *M. alba* and *P. odoratum*. A total of 75 Thai perimenopausal and postmenopausal women aged between 45 and 60 years old (<5 years of menstruation cessation) who lived in northeastern Thailand were enrolled to participate in this study. The recruitment procedures were performed through advertisements at the menopause clinic at Srinagarind Hospital, Faculty of Medicine, Khon Kaen University, Thailand [39]. All participants in this study were women aged between 45 and 60 years old. They were free from serious physical illness such as diabetes, hypertension, allergies, and disorders of the heart, liver, kidney, lung, and mind. Within 3 months of participating in this study, they did not consume any medicines or hormones that affect the function of the nervous system. It was ensured that all subjects did not have alcohol addiction or drug abuse history and did not smoke more than 10 pieces/day. Participants were excluded on the basis of the following criteria: athletes, serious illness, smoking, and alcohol addiction. In addition, subjects who simultaneously participated in other intervention trials were also excluded. In total, 75 subjects were enrolled to participate in this study. Thirty subjects who did not meet the inclusion criteria were excluded, and 45 participants who met all inclusion criteria were subjected to an interview through a semi-structured questionnaire and a physical examination. Written consent was obtained prior to participation in this study.

**Figure 1:**
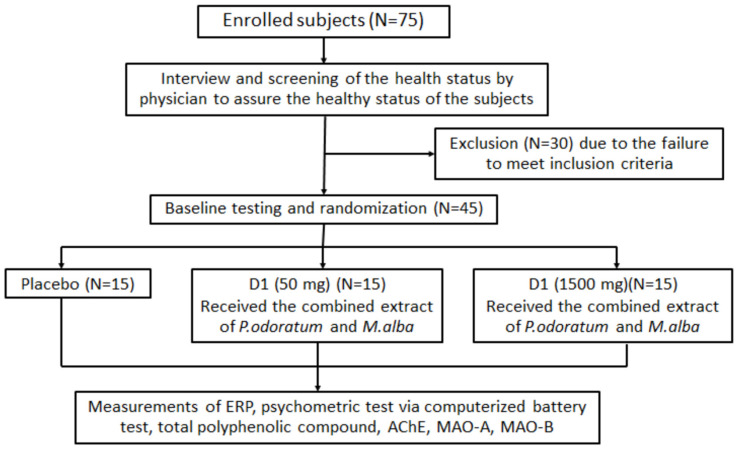
Schematic diagram illustrating experimental protocol.

Using stratified randomization, all participants were allocated to the placebo group, the D1-treated group (functional ingredient 50 mg), or the D2-treated group (functional ingredient 1500 mg). To avoid confounding errors, it was ensured that tea, coffee, and alcoholic beverages were not consumed at least 12 h before the appointment on the experimental days. For 8 weeks, each intervention group was instructed to consume the assigned substance once daily in the morning before breakfast. Prior to the administration of the assigned substances, all subjects had their demographic data measured to ensure that there were no significant differences in any factors that can produce the confounding errors. In addition, the memory condition, attention, and functions of cholinergic and monoamine systems were evaluated by measuring parameters such as event-related potential (ERP), working memory, the activities of acetylcholinesterase (AChE), and both monoamine oxidase type A and type B (MAO-A, MAO-B), and collected as baseline data. Then, at the end of the 8-week study period, all parameters mentioned earlier except demographic data and total phenolic compounds concentrations in serum were monitored. The sequences of all assessment tests were kept constant for all the volunteers. The schematic diagram illustrating the experimental protocol is shown in Figure 1. The instructions for the tests were explained to the volunteers before conducting the study. All volunteers were kept in the dark about whether they were taking D1 (MP 50 mg/serving), D2 (MP 1500 mg/serving), or placebo. The code numbers and the group allocation were revealed only after the assessment of the last subject. All volunteers were instructed to call the study center in case of any adverse effects during the study. The volunteers had the option to withdraw from the study at any time. However, throughout the study period, no subject withdrew. All volunteers were contacted at definite intervals to ensure that they consumed the assigned substance regularly.

### 2.3. Outcome Measurements

#### 2.3.1. Event-Related Potential

Due to the high validity and low bias of the objective data [41], we used objective data such as ERP, a validated factor indicating working memory [13], as the primary outcome. Brain activity was measured using Ag-AgCl disk electrodes on the scalp, according to the international 10/20 system, with reference to linked earlobes using a 40-channel electrode cap (Neuroscan, Inc., Sterling, FL, USA). The detailed procedures are mentioned elsewhere [42]. In brief, the subjects must differentiate between two tones of auditory stimuli (650 Hz and 1 kHz) that served as targeted and non-targeted stimuli delivered at 60 dB via a headphone with a 1250 msec interstimulus interval. The non-targeted tone was provided 85% of total stimuli, whereas the targeted tone was provided 15% of total stimuli. The participants were informed to respond to each tone by pressing the response button in front of them. Using Scan 4.3 analysis software (Neuroscan, Inc., Sterling, FL, USA), the analysis was performed based on changes in latency and peak amplitude of N100 and P300 at Cz locations, which showed the optimum peak patterns [39]. The epochs were extracted from the EEG-free artifact beginning with a 100 msec pre-stimulus and ending with a 500 msec post-stimulus. The baseline correction was applied to each epoch, and voltage changes below 0.1 μV or above 50 μV were excluded from the analysis. N100 was defined as a negative peak that appeared between 65 and 135 msec, whereas P300 was defined as a positive peak that appeared between 280 and 350 msec.

#### 2.3.2. Working Memory Assessment

In this study, four domains of working memory, consisting of power of attention, continuity of attention, quality of memory, and speed of memory, were assessed using a computerized battery test as described elsewhere [16,43,44]. The test-retest reliability in each domain of working memory varied between 0.72 and 0.82 (power of attention = 0.75, continuity of attention = 0.78, quality of working memory = 0.82, and speed of memory = 0.72). A selection of computer-controlled tasks from the system was administered, and the tasks were presented on VGA color monitors for 20 min. All responses were recorded via two-button (YES/NO) response boxes. The computerized battery test was administered to all subjects in the following order.

##### Word Presentation

The subjects were given a 15-word test that was matched for frequency and concreteness via a monitor with a stimulus duration and an interstimulus interval of 1 s. They had to recall each sequence in the correct order.

##### Picture Presentation

The participants were shown a set of 20 photographic images. They were displayed on the monitor at a rate of 1 every 3 s, with a stimulus duration of 1 s. They had to recall the presentation.

##### Simple Reaction Time

In this test, the subjects were exposed to a set of 15 stimuli with an interstimulus interval that varied randomly between 1 and 3.5 s. They were instructed to press the “yes” response button as quickly as possible every time the word “yes” was displayed on the monitor, and their reaction time was recorded.

##### Digit Vigilance Task

During the vigilance test, a target digit was randomly selected and constantly displayed to the right of the monitor screen. A series of digits were displayed in the center of the screen at a rate of 80 /min, and the participant had to press the “yes” button as quickly as possible every time the digit in the series matched the target digit. Both accuracy (%) and reaction time (milliseconds) were recorded.

##### Choice Reaction Time

The subjects were shown the word “no” or “yes” on a monitor, and they had to press the corresponding button as quickly as possible. Both a reaction time (millisecond) and accuracy (%) were recorded. In this test, there were 50 trials, in which the stimulus word was chosen randomly with equal probability, with a randomly varying interstimulus interval of 1 to 3.5 s.

##### Spatial Working Memory

On a motor screen, the participants were shown a picture of a house with four of its nine windows lit. They had to recall the position of the illuminated windows and match it to a subsequent presentation of 36 pictures of the house. They had to respond by pressing the “yes” or “no” response button as quickly as possible. Both reaction time and accuracy were recorded.

##### Numeric Working Memory

The subjects were instructed to memorize a 5-digit stimuli from the presentation and to match them with the new set of 30 probe digits. The response must be given by pressing the “yes” or “no” response button as quickly as possible. This process was repeated two times with different stimuli and probe digits. The mean of both reaction times and the accuracy of responses were recorded.

According to the aforementioned tasks, power of attention was derived from the response time of simple reaction time, choice reaction time, and digit vigilance, whereas continuity of attention was derived from the accurate responses of choice reaction time and digit vigilance tasks as indices. Quality of memory was obtained from the accurate responses of spatial working memory, numeric working memory, word recognition, picture recognition, and spatial memory, while the speed of memory was derived from response times of numeric and spatial working memory as well as word and picture recognition tasks.

### 2.4. Biochemical Assessments

#### 2.4.1. Acetylcholinesterase (AChE) Activity Assessment

The AChE activity of the supplement was determined using a modified Ellman’s method [45]. In brief, the reaction mixture containing 25 µL of 15 mM ATCI, 75 µL of 3 mM DTNB, and 50 µL of 50 mM Tris-HCl, pH 8.0, containing 0.1% bovine serum albumin (BSA) was put into a 96-well microplate and mixed with an aliquot of the tested substance at a volume of 25 µL. Then, the mixture was subjected to a 5 min incubation period. At the end of the incubation period, absorbance was measured at 415 nm (iMark™ Microplate Absorbance Reader). Then, 10 µL of acetylcholine thiochloride (ACTI) was added and incubated for 3 min. The absorbance of the mixture was measured at 415 nm and activity was calculated according to the equation below and expressed as mmol/min.g protein:

AChE activity = (rA/1.36 × 104) × 1/(20/230)C

(Ra = difference in absorbance/minute, C = protein concentration of brain homogenate).

#### 2.4.2. Monoamine Oxidase (MAO) Assessment

Both monoamine oxidase type A and type B (MAO-A, and MAO-B) were measured based on the principle that an amino substrate is converted by MAO into aldehyde, amine, and hydrogen peroxide, which then oxidize 4-aminoantipyrine in the presence of peroxidase. The oxidized 4-aminoantipyrine is condensed with vanillic acid to give a red quinoneimine dye, which can be measured at 498 nm. For the assessment of MAO-A, 50 µL of the sample and 50 µL of 500 nM pargyline were mixed and incubated for 30 min. Then, 200 µL of P-tyramine 500 µM was added, and the absorbance was measured at 490 nm. In order to assess MAO-B activity, all procedures were performed, with the exception of 50 µL of 500 nM chogyline being replaced with 500 nM pargyline [46,47].

### 2.5. Determination of Total Phenolic Compounds

The serum phenolic compound was assessed using the Folin-Ciocalteu colorimetric method, which has been described elsewhere [48]. In brief, an aliquot of 20 µL of the tested sample was mixed with 0.2 mL of Folin-Ciocalteu reagent and 2 mL of distilled water. Then, the mixture was incubated at room temperature for 5 min. At the end of the incubation period, 1 mL of 20% sodium carbonate was added and incubated at room temperature for 2 h. The total polyphenolic compounds were determined using a spectrophotometer to measure the absorbance at 765 nm. Gallic acid was used as a standard and the total phenolics were expressed as gallic acid equivalents (mg/L GAE/mg extract). All measurements were made in triplicate.

### 2.6. Statistical Analysis

Sample size calculations were performed to test the difference between each experimental group and placebo. An evaluable sample size of 15 subjects per group was expected to provide 80% power (two-sided, a = 0.05) based on the ability to detect a 10% difference in brain wave activity, which was used as a primary outcome.

All data were expressed as mean ± SD. An intent-to-treat analysis was performed. Differences in all data were analyzed via analysis of variance. A non-parametric test was used for the analysis of data that failed to show normal distribution. In all statistical comparisons, differences with *p*-value < 0.05 were considered significant.

## 3. Results

### 3.1. Demographic Data of Subjects

The baseline data of all participants are shown in Table 1. No significant differences were observed among different intervention groups.

### 3.2. Changes in Event-Related Potential

**Figure 2:**
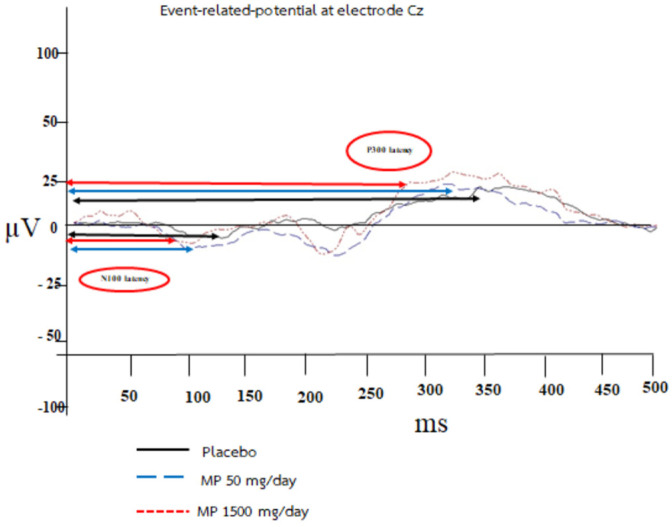
Representative image of event-related potential (ERP) at Cz location showing the latency and amplitude of N100 and P300 waves of the subjects who received the placebo and MP at the doses of 50 and 1500 mg/day.

The effects of various doses of the supplement containing the combined extract of *M. alba* leaves and *P. odoratum* (MP) on the late component of ERP are shown in Figure 2 and Table 2. It was found that after 8 weeks of consumption, the volunteers who consumed the supplement with the functional ingredient (MP) at a dose of 1500 mg per day showed a reduction in N100 and P300 latencies as well as an increase in the amplitudes of both waves (*p*-value < 0.01 all; *p*-value < 0.001 all; compared to the placebo-treated group).

### 3.3. Working Memory Changes

Table 3 shows that the subjects who consumed the supplement containing the functional ingredient (MP) at a dose of 1500 mg per day significantly increased their % response accuracy in the delayed word recognition test, picture recognition test, and spatial memory test (*p*-value < 0.05 all; compared to the placebo group). The volunteers who consumed the supplement containing the functional ingredient at a dose of 1500 mg (*p*-value < 0.05 all; compared to the placebo group) showed a decrease in response time in the delayed word recognition test, simple reaction time test, digit vigilance test, numeric recognition test, picture recognition test, and spatial memory test. Furthermore, the subjects who consumed MP at a dose of 50 mg per day also showed a significant reduction in reaction time in delayed word recognition and simple reaction time test but an increase in % response accuracy of the picture recognition test at the end of the study period (*p*-value < 0.05 all; compared to the placebo group).

### 3.4. Changes in Cholinergic and Monoaminergic Functions

**Figure 3:**
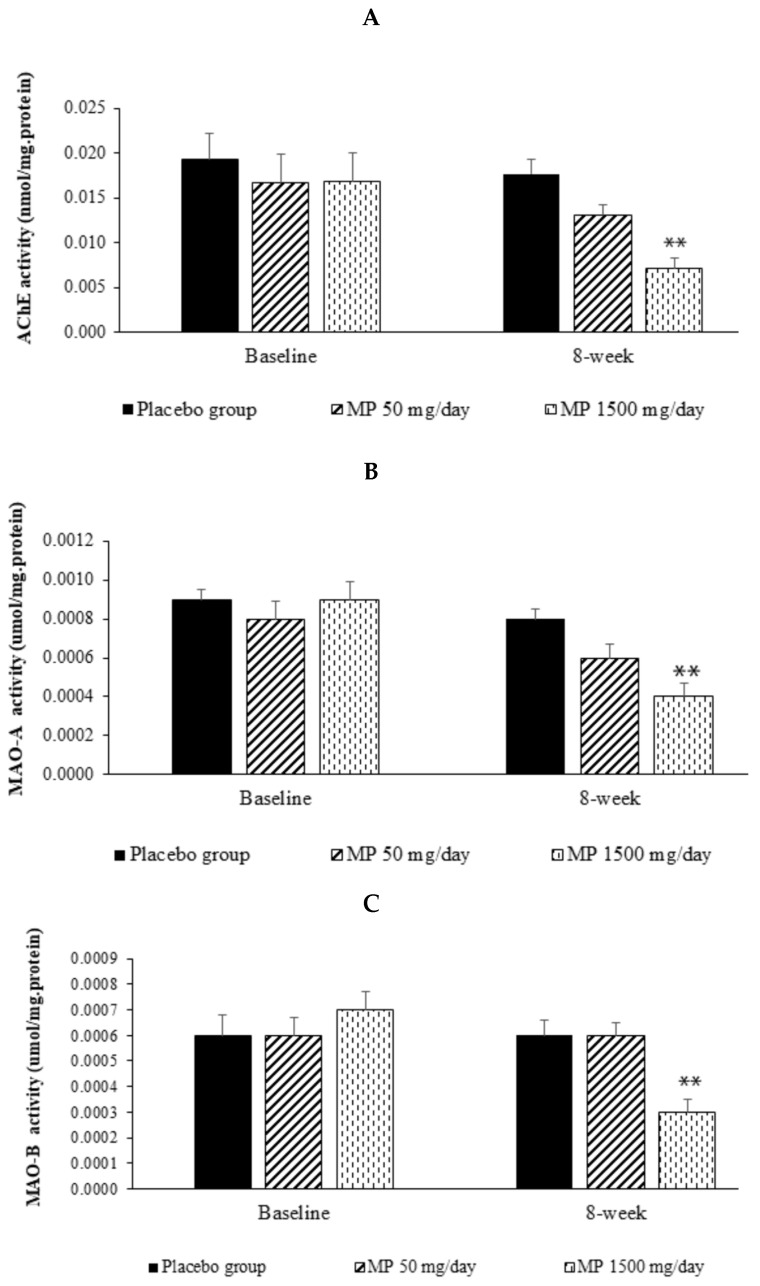
The effect of various doses of the developed supplement containing the combined extract of *Polygonum odoratum* and *Morus alba* leaves on the activities of the neurotransmitter inactivation enzymes in the subjects who consumed the assigned substances at the end of the 8-week study period. (**A**) Acetylcholinesterase (AChE). (**B**) Monoamine oxidase type A (MAO-A). (**C**) Monoamine oxidase type B (MAO-B) (N = 15/group). Values are expressed as mean ± SD. Data are analyzed using ANOVA. ** *p*-value < 0.01; compared to the placebo group.

The possible underlying mechanisms for the positive modulation effect of the developed herbal congee were also investigated. The serum activities of AChE and both types of MAO, the indirect indicators of the cholinergic and monoaminergic functions, were determined. The data are shown in Figure 3. It was found that prior to the interventions, no significant differences were observed in the activities of AChE, MAO-A, and MAO-B in serum of the placebo and both intervention groups. However, the subjects who consumed the supplement containing the combined extract of *P. odorontum* and *M. alba* (MP) at a dose of 1500 mg per day showed a significant reduction in the activities of AChE, MAO-A, and MAO-B in serum (*p*-value < 0.01 all; compared to the placebo-treated group).

### 3.5. Serum Level of Total Phenolic Compounds

**Figure 4:**
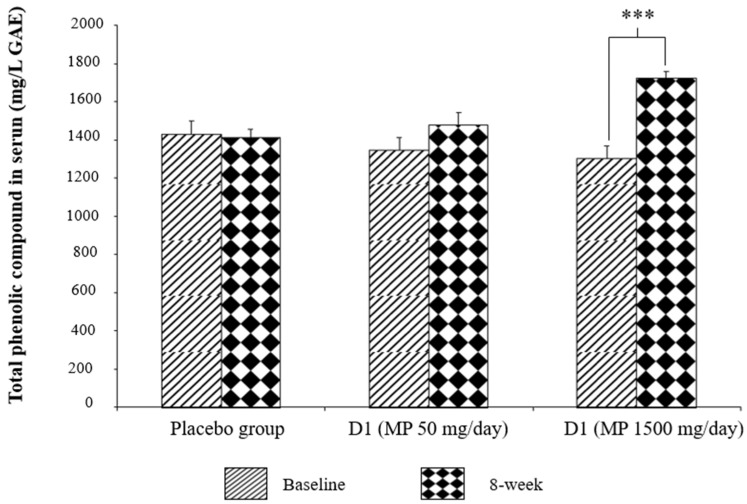
Serum level of the total phenolic compounds in the subjects who consumed the assigned substances at the end of the 8-week study period. (N = 15/group). Values are expressed as mean ± SD. Data are analyzed using ANOVA. *** *p*-value < 0.001; compared to the placebo group.

Figure 4 shows the serum levels of total phenolic compounds of the subjects in all intervention groups. It was shown that the serum level of the total phenolic compounds in the subjects who consumed the supplement containing *P. odoratum* and *M. alba* at a dose of 1500 mg per day was significantly higher than that of the placebo group (*p*-value < 0.001; compared to the placebo-treated group). No significant difference in this parameter was observed in the subjects who consumed a low dose (50 mg) of the supplement containing the combined extract of *P. odoratum* and *M. alba*.

## 4. Discussion

The current study clearly demonstrates that the developed supplement containing the combined extract of *P. odoratum* and *M. alba* at a dose of 1500 mg per day exhibits a cognition-enhancing effect by increasing the amplitudes of both N100 and P300 but decreasing the N100 latency. The subjects in this group also have an increased percentage of accuracy response in the word recognition test, picture recognition test, and spatial memory test. In addition, the subjects in this group also show an increase in the percentage of accuracy response and a decrease in reaction time in the digit vigilance test. Moreover, they also have decreased activities of AChE, MAO-A, and MAO-B in serum.

The current study clearly demonstrates that subjects who consumed the supplement with the functional ingredient at a dose of 1500 mg per day show an increase in accuracy response in delayed word recognition, picture recognition, and spatial memory tests, the tests commonly used for motoring recognition working memory [49,50]. According to the tests mentioned, the volunteers must hold and retrieve stored information. Thus, these data indicate that this group of volunteers show an improvement in the quality of working memory [51], which in turn reflects an improvement in cognitive abilities [52]. Furthermore, our data also reveal an accuracy response elevation and response time reduction in the digit vigilance task, a validity test for assessing attention [53,54,55,56]. Therefore, these data point out that the supplement containing the functional ingredient at a dose of 1500 mg per day can enhance attention and working memory, which in turn indirectly indicates an improvement in cognitive processing abilities.

To ensure the positive modulation effect of the supplement containing the functional ingredient in this study, we also monitored both attention and cognitive processing. It has been revealed that P300 is a sensitive tool for measuring cognitive deterioration [57,58]. Due to the correlation between cognitive impairment and modification in P300, it can be used to measure the efficacy of various treatments on cognitive function [13,59,60]. N100 in ERP is also used to reflect attention [61,62,63,64]. It indicates the initial extraction of information from the sensory analysis of stimulus, which involves attention [65,66]. Our data demonstrate that the volunteers who consumed the supplement containing the functional ingredient at a dose of 1500 mg per day showed a decrease in latency but an increase in the amplitudes of N100 and P300. These findings reflect the improvement in attention and cognitive processing induced by the investigated substance. Furthermore, the findings observed in event-related potential also correspond with the changes obtained from computerized battery tests. Therefore, our data clearly demonstrate that the supplement containing the functional ingredient at a dose of 1500 mg can improve attention to stimuli and cognitive processing, which in turn improve working memory.

To explore the possible underlying mechanism of the developed product, we also determined the effect of the product consumption on the alterations of the cholinergic and monoaminergic systems, which play crucial roles in memory processes [44,67,68,69,70,71], indirectly via the suppression of AChE, MAO-A, and MAO-B, the enzymes that play crucial roles in the inactivation of cholinergic and monoaminergic neurotransmitters [72,73]. It was shown that there is a reduction in AChE, MAO-A, and MAO-B in the volunteers who consumed the supplement containing the functional ingredient at a dose of 1500 mg per day. These findings suggest an increase in cholinergic and monoaminergic functions. Owing to the pivotal roles of both cholinergic and monoaminergic transmitters on memory, particularly attention and cognitive processing, as mentioned earlier, we suggest that the memory enhancement observed in this study may be associated partly with an increase in the functions of the cholinergic system and the monoaminergic system such as serotonin and dopamine due to the suppression of the inactivation processes of acetylcholine and monoamine transmitters, leading to an improvement in synaptic transmission and synchronization of neuronal populations in attention and cognitive processing circuits, resulting in an improvement in working memory [44,70,74,75]. These changes are manifested by the improvement in recognition tests such as word recognition, picture recognition, and spatial memory tests, as well as a reduction in the latencies but an increase in the amplitudes of N100 and P300 brain waves.

Our data correspond to a previous study that shows the cognition-enhancing effect of polyphenol-rich substances in young and middle-aged adults [76] and the cognition-enhancing effect of quercetin-enriched onion [77]. The current data reveal that the total polyphenolic compound content in the serum of volunteers of other groups fail to show a significant change at the end of the study, whereas the subjects in the high dose treatment group show a significant elevation in serum. Therefore, the positive modulation effect on memory observed in this study may possibly relate to the polyphenolic compound content, such as quercetin, present in the supplement containing the functional ingredient derived from the combined extract of *M. alba* and *P. odoratum*.

The major strength of our study is its randomized, double-blind, placebo-controlled, parallel-group design. Compliance with the consumption of the developed product was 100% in all groups. In addition, we use the brain wave, validated objective data, together with a battery of computerized psychometric tests. This study provides a validated and sensitive tool for assessing the working memory of Thai menopausal and elderly women to ensure the positive modulation effect of the developed functional ingredient. The possible mechanism of the developed product is also explored in parallel with the cognitive function assessment in order to avoid other confounding errors such as the effect of different environments and emotions. However, a limitation of this study is the difference in lifestyles, environment, physical activity, mood of subject, level of social and cultural interaction, and diet, all of which can modulate the epigenetic mechanism. However, we minimized the influence of polyphenol-enriched food consumption and physical activity by measuring both amount and types of food intake and physical activity per week, and no significant changes were observed.

## 5. Conclusions

This study clearly demonstrates that the functional ingredient developed from the combined extract of *M. alba* and *P. odoratum* significantly increases working memory in menopausal women. The underlying mechanism may occur through an increase in the function of both cholinergic and monoaminergic systems, the neurotransmitters that play a crucial role in the memory process.

## Figures and Tables

**Table 1:** The demographic data of subjects.

Parameters	Placebo	MP 50 mg/Day	MP 1500 mg/Day
Age (years)	51.41 ± 4.21	50.47 ± 3.20 F(1,28) = 0.536 P = 0.470	50.47 ± 3.64 F(1,28) = 0.484 P = 0.492
Education (years)	7.73 ± 4.89	7.73 ± 4.13 F(1,28) = 0.000 P = 1.000	7.47 ± 4.50 F(1,28) = 0.024 P = 0.878
Body mass index	24.27 ± 2.91	25.23 ± 3.52 F(1,28) = 0.652 P = 0.426	24.91 ± 3.81 F(1,28) = 0.269 P = 0.608
Blood sugar	87.33 ± 13.50	84.67 ± 6.89 F(1,28) = 0.464 P = 0.501	88.93 ± 15.04 F(1,28) = 0.094 P = 0.761
Uric acid	5.34 ± 0.77	5.05 ± 0.76 F(1,28) = 1.097 P = 0.304	5.43 ± 0.92 F(1,28) = 0.078 P = 0.782
Heart rate	77.00 ± 12.86	73.20 ± 9.20 F(1,28) = 1.269 P = 0.269	74.27 ± 8.66 F(1,28) = 0.779 P = 0.385
Respiratory rate	19.80 ± 2.40	18.00 ± 1.98 F(1,28) = 1.063 P = 0.332	18.80 ± 1.82 F(1,28) = 1.656 P = 0.209
Systolic blood pressure (mmHg)	115.80 ± 11.48	118.67 ± 13.84 F(1,28) = 0.381 P = 0.542	119.13 ± 13.06 F(1,28) = 0.551 P = 0.464
Diastolic blood pressure (mmHg)	75.87 ± 8.93	78.00 ± 9.38 F(1,28) = 0.407 P = 0.529	76.33 ± 8.47 F(1,28) = 0.022 P = 0.884

Values are presented as mean ± standard deviation. (N = 15/group).

**Table 2:** The effect of various doses of the developed supplement containing the combined extract of *Polygonum odoratum* and *Morus alba* leaves on event-related potential (ERP).

Brain Wave	Group	Baseline	8 Weeks
N100 amplitude (μV)	Placebo	12.85 ± 7.86	13.59 ± 4.52
	MP 50 mg/day	9.93 ± 6.77 F(1,28) = 1.601 P = 0.206	14.35 ± 5.24 F(1,28) = 0.589 P = 0.443
	MP 1500 mg/day	9.38 ± 6.76 F(1,28) = 2.201 P = 0.333	20.06 ± 6.43 ** F(1,28) = 10.808 P = 0.004
N100 latency (msec)	Placebo	111.47 ± 23.26	102.47 ± 13.26
	MP 50 mg/day	110.07 ± 25.59 F(1,28) = 0.025 P = 0.877	98.73 ± 18.35 F(1,28) = 0.408 P = 0.528
	MP 1500 mg/day	108.00 ± 22.65 F(1,28) = 0.171 P = 0.682	75.40 ± 6.78 *** F(1,28) = 49.575 P < 0.001
P300 amplitude (UV)	Placebo	17.54 ± 10.31	16.94 ±7.20
	MP 50 mg/day	16.69 ± 5.74 F(1,28) = 1.708 P = 0.191	20.14 ± 7.56 F(1,28) = 3.806 P = 0.051
	MP 1500 mg/day	14.65 ± 7.40 F(1,28) = 2.235 P = 0.327	26.28 ± 6.15 *** F(1,28) = 23.847 P < 0.001
P300 latency (msec)	Placebo	317.87 ± 32.54	323.20 ± 23.12
	MP 50 mg/day	328.87 ± 16.47 F(1,28) = 0.190 P = 0.663	307.47 ± 14.66 F(1,28) = 1.447 P = 0.229
	MP 1500 mg/day	330.93 ± 32.08 F(1,28) = 0.379 P = 0.827	280.40 ± 11.82 ** F(1,28) = 11.248 P = 0.004

Values are expressed as mean ± SD (N = 15/group). Data are analyzed using ANOVA. **, *** *p*-value < 0.01, 0.001, respectively, compared to the placebo group.

**Table 3:** The effect of various doses of the developed supplement containing the combined extract of *Polygonum odoratum* and *Morus alba* leaves on working memory assessed using computerized battery tests.

Parameters	Groups	Baseline	8 Weeks
(1) Delayed word recognition (% accuracy)	Group 1: Placebo Group 2: MP 50 mg/day Group 3: MP 1500 mg/day	81.56 ± 9.33 82.44 ± 14.10 78.89 ± 11.25	84.44 ± 9.65 84.89 ± 9.50 90.66 ± 5.37 *
(2) Delayed word recognition reaction time (msec)	Group 1: Placebo Group 2: MP 50 mg/day Group 3: MP 1500 mg/day	1911.15 ± 474.01 1936.77 ± 627.23 1958.92 ± 721.37	1632.90 ± 650.36 1229.69 ± 239.25 * 1276.61 ± 285.61 *
(3) Simple reaction time (msec)	Group 1: Placebo Group 2: MP 50 mg/day Group 3: MP 1500 mg/day	769.44 ± 206.93 690.37 ± 173.31 761.06 ± 193.27	738.08 ± 246.57 611.36 ± 112.72 * 585.41 ± 60.23 **
(4) Digit vigilance (% accuracy)	Group 1: Placebo Group 2: MP 50 mg/day Group 3: MP 1500 mg/day	55.56 ± 25.66 53.33 ± 24.82 62.22 ± 25.78	66.70 ± 18.86 74.00 ± 14.27 86.81 ± 8.02 ***
(5) Digit vigilance reaction time (msec)	Group 1: Placebo Group 2: MP 50 mg/day Group 3: MP 1500 mg/day	657.91 ± 71.39 666.77± 42.22 647.59± 60.11	641.63 ± 39.38 656.10 ± 61.20 583.35 ± 84.79 *
(6) Choice reaction time (%accuracy)	Group 1: Placebo Group 2: MP 50 mg/day Group 3: MP 1500 mg/day	96.93 ± 6.13 97.60 ± 8.95 97.47 ± 2.56	98.80 ± 1.66 98.67 ± 1.63 98.27 ± 1.49
(7) Choice reaction time response (msec)	Group 1: Placebo Group 2: MP 50 mg/day Group 3: MP 1500 mg/day	888.53 ± 122.17 946.71 ± 139.12 985.10 ± 122.45	875.51 ± 159.11 871.84 ± 112.36 828.72 ± 110.44
(8) Numeric working memory (% accuracy)	Group 1: Placebo Group 2: MP 50 mg/day Group 3: MP 1500 mg/day	90.00 ± 9.76 91.78 ± 10.30 83.53 ± 16.20	91.96 ± 10.51 93.33 ± 8.07 91.11 ± 9.14
(9) Numeric working memory reaction time (msec)	Group 1: Placebo Group 2: MP 50 mg/day Group 3: MP 1500 mg/day	1332.83 ± 296.88 1402.40 ± 333.81 1327.19 ± 318.30	1384.53 ± 558.66 1171.37 ± 320.52 1095.34 ± 129.58 *
(10) Picture recognition (% accuracy)	Group 1: Placebo Group 2: MP 50 mg/day Group 3: MP 1500 mg/day	79.33 ± 7.04 83.00 ± 9.22 78.00 ± 9.41	83.00 ± 10.14 90.67 ± 9.42 * 89.67 ± 5.50 *
(11) Picture recognition reaction time (msec)	Group 1: Placebo Group 2: MP 50 mg/day Group 3: MP 1500 mg/day	2044.87 ± 677.74 1963.24 ± 668.95 1759.14 ± 482.52	1565.55 ± 377.62 1348.75 ± 256.59 1300.18 ± 380.14 *
(12) Spatial working memory (% accuracy)	Group 1: Placebo Group 2: MP 50 mg/day Group 3: MP 1500 mg/day	78.85 ± 19.89 79.81 ± 17.52 72.05 ± 22.64	82.37 ± 14.84 86.21 ± 9.78 92.59 ± 5.00 *
(13) Spatial working memory reaction time (msec)	Group 1: Placebo Group 2: MP 50 mg/day Group 3: MP 1500 mg/day	2052.41 ± 802.14 2037.64 ± 709.31 1659.07 ± 402.16	1778.10 ± 525.19 1570.62 ± 346.19 1411.81 ± 241.06 *

Values are expressed as mean ± SD (N = 15/group). Data are analyzed using ANOVA. *, **, *** *p*-value < 0.05, 0.01, 0.001, respectively, compared to the placebo group.

## Data Availability

The data used to support the findings of this study are available from the corresponding author upon request.
